# Balance and gait disorders in de novo Parkinson’s disease: support for early rehabilitation

**DOI:** 10.1007/s00415-024-12804-4

**Published:** 2024-12-12

**Authors:** Beata Lindholm, Peter Hagell, Per Odin, Oskar Hansson, Arkadiusz Siennicki-Lantz, Sölve Elmståhl, Lars B. Dahlin, Erika Franzén

**Affiliations:** 1 Cognitive Disorders Unit, Department of Clinical Sciences Malmö, Lund University, 205 02 Malmö, Sweden; 2 Department of Neurology, Rehabilitation Medicine, Memory Disorders and Geriatrics, Skåne University Hospital Malmö, 205 02 Malmö, Sweden; 3 Department of Clinical Sciences in Malmö, Division of Geriatric Medicine, Lund University, Malmö, Sweden; 4 The PRO–CARE Group, Faculty of Health Sciences, Kristianstad University, 291 39 Kristianstad, Sweden; 5 Division of Neurology, Department of Clinical Sciences Lund, Lund University, Lund, Sweden; 6 Department Translational Medicine, Lund University, Malmö, Sweden; 7 Department of Hand Surgery, Skåne University Hospital, Malmö, Sweden; 8 Department of Biomedical and Clinical Sciences, Linköping University, Linköping, Sweden; 9 Division of Physiotherapy, Department of Neurobiology, Care Sciences and Society, Karolinska Institutet, Huddinge, Sweden; 10 Medical Unit Allied Health Professionals, Women’s Health and Allied Health Professionals Theme, Karolinska University Hospital, Stockholm, Sweden

**Keywords:** Parkinson’s disease, De novo, Balance and gait, Falls, Near falls, Neurorehabilitation

## Abstract

**Background:**

Postural instability is considered a late complication of Parkinson’s disease (PD). However, growing evidence shows that balance and gait problems may occur early in the disease.

**Objective:**

To describe balance, gait, and falls/near falls in persons with newly diagnosed, untreated PD (“de novo”), and to compare this with persons with mild-moderate PD (Later PD). In addition, we evaluated differences relative to PD subtypes in de novo PD.

**Methods:**

De novo (*n* = 54) and Later (*n* = 58) PD were assessed regarding motor symptoms, balance, gait, and falls/near falls.

**Results:**

At least 25% of de novo PD had impaired reactive balance and/or comfortable gait speed ≤ 1.0 m/s. At least 50% had abnormal dynamic balance. A third reported balance problems during dual-tasking. Five persons (9%) reported falls/near falls. The median (*q*1–*q*3) motor symptom score was 21 (14–28) in de novo PD and 13.5 (9–20) in Later PD (*p* < 0.001). Later PD performed worse on more balance-demanding tests and a higher percentage of individuals reported falls/near falls (*p* ≤ 0.048). De novo PIGD PD (*n* = 10) exhibited worse motor symptoms, reactive and dynamic balance, gait speed, mobility, and freezing of gait as compared to the non-PIGD de novo PD (*n* = 37) (*p* ≤ 0.049).

**Conclusion:**

Balance and gait were impaired in de novo PD and most pronounced in PIGD subtype. In addition, balance difficulties during dual-tasking and falls/near falls were evident during this early stage. The lower scores of motor symptoms in Later PD did not result in better mobility, balance, or less falls/near falls indicating that medications have less effect on these symptoms.

## Introduction

Postural instability is a cardinal sign of Parkinson’s disease (PD), and balance and gait difficulties are among the most distressing symptoms of PD [3]. There is growing evidence that balance and gait difficulties as well as falls may occur early in the course of the disease, sometimes already in individuals with newly diagnosed, untreated PD (“de novo”) [6, 12, 28, 29, 32, 34, 62, 63].

Everyday transfers and activities (e.g., walking, turning, and transferring to/from sitting) induce self-generated perturbations that challenge balance. Walking is particularly challenging because the body is in a continuous state of imbalance, and the only way to prevent falling is to take the next step. PD-related walking difficulties are characterized by decreased gait speed, reduced step length, shuffling and turning difficulties, as well as freezing of gait (FOG). Walking may be affected by conducting motor and cognitive dual tasks [31]. In addition, stooped posture, reduced arm swing [31], and striatal foot deformities may aggravate balance and gait deficit [36].

It is also well-known that falls are one of the most disabling features of PD and become more common during the disease. History of falls is often suggested as the strongest significant predictor of future falls [7, 31, 46]. However, this is of limited use when aiming to work proactively. Therefore, we have previously proposed to consider near falls that often precede actual falls. In fact, near falls have turned out to be the strongest predictor of future falls [39]. Most falls occur while walking, but also while turning, transferring to/from sitting, bending forwards, or reaching [31]. This highlights the importance of using assessments that mimic daily tasks and activities and target various aspects of balance and gait [40, 44, 53, 61].

Based on the presence of prominent motor symptoms investigated with the Unified Parkinson’s Disease Rating Scale (UPDRS) [14] it is, by definition, stated that persons with the PD subtype Postural Instability and Gait Difficulty (PIGD) suffer from more severe balance and gait difficulties than the tremor dominant (TD) subtype [26]. The key components of balance and gait evaluation according to UPDRS include assessment of reactive balance and ability to walk.

In this study, we aimed to describe various aspects of balance and gait as well as falls and near falls in persons with de novo PD and to compare this with persons with mild-moderate PD. In addition, we evaluated differences relative to PD subtypes in persons with de novo PD.

## Methods

### Participants and assessments

All participants received care at a neurology outpatient clinic in southern Sweden and were consecutively recruited by a movement disorder specialist as a part of usual clinical care.

Sixty-nine persons with suspected drug-naive PD were identified and considered for inclusion in the study during the years 2019–2021. Those who were unable to understand instructions (*n* = 5) or had severe comorbidity (*n* = 2) were excluded. Eight persons declined to participate. The final study sample included 54 persons, hereafter called de novo* PD*. The diagnosis was made according to the Movement Disorders Society (MDS) diagnostic criteria [54]. The decision to start anti-parkinsonian therapy involves consideration of individual symptoms and needs, and therefore could occur at different time-points after diagnosis.

The de novo PD cohort was compared with an existing cohort collected during the years 2012–2017 for other purposes, using the same exclusion criteria [38]. This cohort (*n* = 58; hereafter called *Later PD*) consisted of people with a clinically established PD diagnosis according to United Kingdom Parkinson’s Disease Society Brain Bank criteria [24]. The mean (SD) disease duration of Later PD cohort was 6.7 (3.7) years, and the median (*q*1–*q*3; min–max) daily levodopa equivalent (LDE) dose [60] was 500 (400–675, 150–1580) mg/day. At the time of assessments, 48 out of the 58 participants (83%) self-rated their motor status as “good”, whereas 10 (17%) rated it as “bad”. More detailed descriptions of methods for recruitment of this cohort are available elsewhere [37, 38].

All assessments in both cohorts were conducted by the same specialist in neurological physiotherapy with extensive experience of PD (BL) during outpatient visits which were scheduled at a time of day when the participant usually reported feeling at his/her best. An overview of the assessments is presented in Table 1. Assessments targeted motor symptoms [14], activity of daily living [14], disease severity staging [21], cognition [10, 13], as well as various aspects of balance and gait. The clinical test battery included evaluation of reactive balance (Nutt Retropulsion Test, NRT) [61] and dynamic balance (tandem gait test, TG [37, 45] mobility (timed up and go test, TUG) [53] as well as comfortable and fast gait speed (10-Meter Walk Test, 10MWT [40]). In addition, the participants completed questionnaires targeting balance problems while dual-tasking [39], FOG [50], and walking ability [4]. As a last step of the outpatient visits, participants were asked to keep a diary for prospective recording of falls and near falls for 3 months. Falls were defined as “an unexpected event in which the person comes to rest on the ground, floor, or lower level” [39]. Near falls were defined as “a fall initiated but arrested by support from the wall, railing, other person, etc.” [39].

**Table 1:** Included assessments in both cohort

Assessment	Score range	References
Motor symptoms (UPDRS part III)	0–108^a^	[14]
Activities of daily living (UPDRS part II)^b^	0–52^a^	[14]
Disease severity (H&Y)	I–V^a^	[21]
Cognition (MOCA)	0–30^c^	[10]
Executive function (FAB)	0–18^c^	[13]
Reactive balance (NRT) Sudden, unexpected backward pull to the shoulders from behind when standing with feet slightly apart and eyes open	0 (normal, may take 2 steps to recover) 1 (retropulsion, take 3 or more steps; recovers unaided) 2 (would fall if not caught) 3 (spontaneous tendency to fall or unable to stand unaided, test not executable)	[61]
Dynamic balance (TG) Ten heel-to-toe steps along a straight line, with eyes open and without walking aids/support (those using walking aids were asked to try to perform TG without support)	0 (no sidesteps) 1 (single sidestep) 2 (multiple sidesteps) 3 (unable to take 4 consecutive steps)	[37, 44]
Mobility (TUG) Time (s) to rise from an armchair, walk at a comfortable and safe pace to a line on the floor three meters away, turn and walk back to the chair and sit down again	≥ 0 s^a^	[53]
Dual tasking difficulties Do you experience balance problems while standing or walking when doing more than one thing at a time, e.g., carrying a tray while walking?	No/yes	[39]
Comfortable gait speed (CGS,10MWT) Fast gait speed (FGS, 10MWT)	≥ 0 m/s^c^	[40]
Time (s) to walk 10 m (without acceleration/deceleration distance) in comfortable and fast gait speed, respectively		
Freezing of gait (FOG, item 3, FOGQsa) Do you feel that your feet get glued to the floor while walking, making a turn or when trying to initiate walking?	0 (never) 1 (very rarely—about once a month) 2 (rarely—about once a week) 3 (often—about once a day) 4 (always—whenever walking)	[50]
Self-reported walking ability (Walk–12G)	0–42^a^	[4]
Falls during three months follow-up Did you fall in such a way that your body hit the ground?	No/yes	[39]
Near falls during three months follow-up Were you close to falling, but managed to brace yourself at the last moment (e.g., grabbed on to someone, to an object or the wall?	No/yes	[39]

*FOGQsa* FOG Questionnaire, self–administered version; *H&Y* Hoehn and Yahr stage, *MOCA* Montreal Cognitive Assessment, *FAB* frontal assessment battery, *NRT* Nutt Retropulsion Test, *UPDRS* Unified Parkinson’s Disease Rating Scale, *10MWT* 10-Meter Walk Test, *m/s* meters per second, *TG* Tandem Gait, *TUG* Timed Up and Go, *Walk–12G* 12–item generic walking scale

^a^Higher = worse

^b^Items 13–16 were used to estimate subtypes of PD[16]

^c^Higher = better

### PD subtype classifications

Participants were classified into the PIGD, tremor dominant (TD), and indeterminate (IT) subtypes of PD [57]. The ratio of the mean UPDRS tremor scores (UPDRS III items 20–21 and UPDRS II item 16) to the mean UPDRS PIGD scores (UPDRS III items 29 and 30 and UPDRS II items 13–15) was used to identify PIGD subtype (ratio ≤ 1), TD subtype (ratio ≥ 1.5) and IT subtype (ratios > 1.0 and < 1.5).

### Statistical analysis

IBM SPSS software version 25.0 was used to perform all statistical analyses. Normally distributed interval/ratio level variables were described using means and SDs. In other cases, medians (*q*1–*q*3) were used. Categorical variables were described using n (%). For between-group comparisons, the independent samples *t* test or Mann–Whitney *U* test was used as appropriate, and the Chi-square test was used for categorical variables. The alpha level of significance was set at 0.05 (two tailed).

## Results

### De novo PD cohort

The mean (SD) age of the de novo PD cohort (*n* = 54) was 67 (11.0) years and included 20 (37%) females. The median (*q*1–*q*3, min–max) UPDRS III score was 21 (14–28; 8–53). At least one-fourth of the de novo PD had abnormal reactive balance and needed three or more steps or support to prevent falling during unexpected shoulder pull (NRT). At least half had abnormal dynamic balance and needed to take at least one single sidestep during TG. At least one-fourth of these presented multiple sidesteps or inability to take four consecutive heel-to-toe steps. Fifteen participants (29%) reported balance problems while standing or walking when doing more than one thing at a time, e.g., carrying a tray while walking. At least one-fourth had a comfortable gait speed of ≤ 1.0 m/s. Five participants (9%) reported experience of falls/near falls during the 3-month follow-up. Further details are presented in Table 2.

**Table 2:** Characteristics and comparison of de novo PD vs Later PD

	De novo PD *N* = 54	Later PD *N* = 58	*p* value
Age (years)	67 (11.0; 42–83)	69 (8.9, 46–84)	0.216^d^
Female gender	20 (37)	32 (55)	0.560^c^
Disease severity (HY)^a^	II (II–II; I–IV)	II (II–III; I–IV)	0.114^e^
Cognition (MOCA)^b^	26 (24–27; 20–30)	25 (23–27;14–30)	0.329^e^
Executive function (FAB)^b^	16 (14–16; 3–18)	16 (14–17; 7–18)	0.766^e^
Motor symptoms (UPDRS)^a^	21 (14–28; 8–53)	13.5 (9–20; 1–40)	** < 0.001**^**e**^
Reactive balance (NRT)^a^	0 (0–1; 0–2)	0 (0–1; 0–3)	0.432^e^
Dynamic balance (TG)^a^	1 (0–2; 0–3)	2 (0–3;0–3)	**0.048**^**e**^
Mobility (TUG), s^a^	9.2 (7.7–10.7; 6.4–28.4)	10.5 (8.5–13.1; 6.8–54.1)	**0.013**^**e**^
Dual tasking difficulties	15 (29)^n=51^	29 (52)^n=56^	**0.019**^**c**^
CGS (10MWT), m/s^b^	1.1 (1.0–1.3; 0.5–1.6)	1.2 (0.9–1.3; 0.4–1.7)	0.786^e^
FGS (10MWT), m/s^b^	1.6 (1.3–1.9; 0.6–1.3)	1.5 (1.2–1.8; 0.5–2.3)	0.086^e^
FOG (FOGQsa item 3)^a^	0 (0–0; 0–4)^n=47^	1 (0–3; 0–4)	** < 0.001**^**e**^
Walking ability (Walk12G)^a^	7 (1–16; 0–33)^n=48^	10 (5–24; 0–42)^n=56^	**0.006**^**e**^
Number of individuals with one or more prospective falls	5 (9)	26 (45)	** < 0.001**^**c**^
Number of Individuals with one or more prospective near falls	5 (9)	19 (33)	** < 0.001**^**c**^

Values are mean (SD; min–max), median (*q*1–*q*3; min–max) or n (%). Significant *p* values are indicated in bold

*CGS* comfortable gait speed, *FAB* frontal assessment battery, *FGS* Fast Gait speed, *FOGQsa* Freezing of Gait Questionnaire self-administered version, *H&Y* Hoehn and Yahr stage, *MOCA* Montreal Cognitive Assessment*,* *NRT* Nutt Retropulsion Test, *UPDRS* Unified Parkinson’s Disease Rating Scale, *10MWT* 10-Meter Walk Test, *m/s* meters per second, *TG* Tandem Gait, *TUG* Timed Up and Go, *Walk–12G* 12-item generic walking scale

^a^Higher = worse

^b^Higher = better

^c^Chi-test

^d^Student’s *T* test

^e^Mann–Whitney *U* test

### De novo PD cohort vs Later PD cohort

Comparison between participants with de novo PD (*n* = 54) and Later PD (*n* = 58) did not show any differences regarding age or gender (Table 2). Clinical assessments showed that participants with de novo PD had more motor symptoms but performed better on dynamic balance (TG) and mobility (TUG) tests than those with Later PD. De novo PD also had better self-reported walking ability, less FOG, and dual-tasking difficulties, and a lower percentage of individuals with falls/near falls. There were no differences between de novo and Later PD regarding reactive balance and comfortable or fast gait speed. Further details are presented in Table 2.

### De novo PIGD PD cohort vs de novo non-PIGD cohort

The PD subtype could not be determined for 7 of 54 participants due to missing data. Ten of the remaining forty-seven participants were identified as PIGD, thirty-six as TD, and one participant as IT subtype. Due to relatively small samples, the IT and TD subtypes were combined to form a single non–PIGD (*n* = 37) [52].

There were no differences between subtypes regarding age or gender (Table 3). However, those with the PIGD subtype exhibited worse motor symptoms, reactive and dynamic balance, mobility, comfortable and fast gait speed, FOG, and self-reported walking ability, as compared to those with the non-PIGD subtype (*p* ≤ 0.049). There were no differences between subtypes regarding dual-tasking difficulties and falls/near falls.

**Table 3:** Comparison of de novo PD with postural instability and gait (PIGD) subtype vs de novo PD with non-PIGD subtype

	De novo PIGD PD *N* = 10	De novo non-PIGD PD *N* = 37	*p* value
Age (years)	69 (5.0; 64–78)	66 (11.7; 45–83)^d^	0.582^d^
Female gender	4 (40)	14 (38)	0.999^c^
Disease severity (HY)^a^	III (II–III; II–III)	II (II–II; I–III)	**0.001**^**e**^
Cognition (MOCA)^b^	24.5 (23–27; 21–29)	26 (24–28; 17–30)	0.487^e^
Executive function (FAB)^b^	14.5 (11–17; 9–17)	16 (15–17; 3–18)	0.241^e^
Motor symptoms (UPDRS)^a^	26 (21–34; 13–42)	18 (14–25; 8–53)	**0.049**^**e**^
Reactive balance (NRT)^a^	2 (0–2; 0–2)	0 (0–0; 0–2)	** < 0.001**^**e**^
Dynamic balance (TG)^a^	3 (1–3; 0–3)	0 (0–1; 0–3)	**0.003**^**e**^
Mobility (TUG), s^a^	10.8 (10.3–12.0; 7.3–15.0)	8.9 (7.6–10.5; 6.4–19.1)	**0.013**^**e**^
Dual tasking difficulties	4 (44) ^n=9^	9 (26) ^n=35^	0.272^c^
CGS (10MWT), m/s^b^	1.0 (0.9–1.0; 0.6–1.1)	1.2 (0.9–1.4; 0.5–1.6)	**0.009**^**e**^
FGS (10MWT), m/s^b^	1.3 (1.3–1.6; 0.8–1.7)	1.7 (1.4–2.1; 0.7–3.1)	**0.020**^**e**^
FOG (FOGQsa item 3)^a^	0 (0–4; 0–4)	0 (0–0; 0–2)	**0.002**^**e**^
Walking ability (Walk12G)^a^	17.5 (13–20; 8–30)	4 (1–12; 0–33)	** < 0.001**^**e**^
Number of individuals with one or more prospective falls	2 (20)	2 (5)	0.142^c^
Number of individuals with one or more prospective near falls	1 (10)	3 (8)	0.849^c^

Values are mean (SD; min–max), median (q1–q3; min–max) or n (%). Significant p-values are indicated in bold

*CGS* comfortable gait speed, *FAB* frontal assessment battery, *FGS* Fast Gait speed, *FOGQsa* Freezing of Gait Questionnaire self-administered version, *H&Y* Hoehn and Yahr stage, *MOCA* Montreal Cognitive Assessment*,* *NRT* Nutt Retropulsion Test, *UPDRS* Unified Parkinson’s Disease Rating Scale, *10MWT* 10-Meter Walk Test, *m/s* meters per second, *TG* Tandem Gait, *TUG* Timed Up and Go, *Walk–12G* 12-item generic walking scale

^a^Higher = worse

^b^Higher = better

^c^Chi–test

^d^Student's *T* test

^e^Mann–Whitney *U* test

## Discussion

We found that various aspects of balance and gait are impaired already in newly diagnosed PD and, as expected, more pronounced among participants with the PIGD subtype. Falls/near falls also occurred early but were not subtype-specific. Although the group with de novo PD showed more motor symptoms than the later-stage cohort, they had better balance and gait ability and less falls/near falls.

Our findings are in line with previous studies highlighting early deficits of balance and gait in PD [6, 12, 28, 29, 32, 34, 62, 63]. For example, longitudinal results suggest that balance impairments exist even at the drug-naive early stages of the disease and worsen during the ensuing 5 years [28]. Despite this growing evidence, recent reviews [2, 59] still describe postural instability and falls as occurring in the more advanced stages of the disease. This may contribute to a delay in participation in neurorehabilitation programs which can lead to increased suffering, accidents, and unnecessary community costs [17]. Meanwhile, and as suggested previously, our findings imply that successful symptomatic medication in the more advanced stages of PD has less effects on demanding aspects of balance and gait [5, 16, 23, 28, 42, 43, 46, 49]. This indicates a need for alternative treatment efforts and screening for fall risk already from an early stage of the disease. In fact, early referral to physiotherapy for examination, advice, and interventions has been recently recommended [16].

A comfortable gait speed of < 1.1 m/s is a well-known independent predictor of future falls in people with PD [31]. At least one-fourth of our de novo PD cohort presented gait speeds below this cutoff value. Similar results for persons with de novo PD have been reported earlier [32, 34]. The reduced gait speed might be due to brady/hypokinesia and can be improved by anti-parkinsonian medication [11, 32]. However, the causal relation between gait speed, motor subtypes, training, and medication should be best studied in a longitudinal study design that preferably commences at the time of diagnosis. However, such studies are resource-demanding and rare.

Our results regarding the occurrence of early deficit in reactive balance are largely in line with previously reported findings [28], Reactive balance according to NRT reflects everyday life situations when the balance is often challenged by unexpected self-perturbation due to, e.g., tripping [61]. Abnormal NRT performance has been suggested as a better predictor of falls in PD than item 30 of the UPDRS which involves an expected shoulder pull [61]. Thus, at least one-fourth of the de novo PD cohort has an increased risk of falling due to everyday self-perturbation. Moreover, a comparison between the de novo PD and Later stages PD did not show differences in NRT scores. This finding is in line with previous studies reporting that postural instability responds poorly to dopaminergic therapy [15, 49].

This study appears to be the first to report on dynamic balance in de novo PD. However, the importance of TG for the investigation of dynamic balance in the later stages of PD has been highlighted before [37, 44, 45]. Abnormal test performance was observed in at least half of our de novo PD cohort. The critical point of tandem gait is the exact foot placement and ability to keep the center of mass within a very narrow base of support [58]. This test challenges mediolateral stability—an important aspect of postural stability in PD that, if impaired, may contribute to falls [22, 30, 37].

Early identification of FOG in PD is of particular importance because of its high association with falls [31]. FOG was reported by some individuals in our de novo cohort. This result is in line with a recently published study that also reported early occurrence of FOG in drug-naive PD [56]. Importantly, according to recent reviews, the frequency of FOG increases as PD progresses and is usually seen in the later stages of the disease [2, 59]. Thus, it is plausible that some of the de novo PD were later in the course of disease than expected at the time of diagnosis. In fact, some individuals in our de novo PD cohort demonstrated impaired cognition which is also attributed to later stages of the disease [2, 59]. Limited access to neurologists and/or delayed referral from health care may be potential explanations. In both cases, the administration of anti-parkinsonian medication and referral to a beyond becomes unnecessarily delayed, which can have negative consequences on activities of everyday life and quality of life. In fact, according to the Swedish National Parkinson's Disease Patient Registry (PARKreg), the time between onset of Parkinsonian symptoms and diagnosis was as long as 1–2 years [19]. However, in some cases, a person could be de novo because of medical or own decision to not start medication. That may contribute to a variety of balance and gait impairments in our de novo PD cohort. Finally, PD diagnosis may be uncertain in the early stages of the disease and misdiagnoses may occur in up to 24% [59]. That speaks again for the importance of early care at the specialist clinic.

Motor symptoms in de novo PD were generally milder than those observed in previous studies [27, 33, 34]. For example, the median UPDRS III score for drug-naive early stages of PD, presented by Johansson et al. [28], was 26, as compared to 21 in our cohort, whereas our cohort was younger than that of Johansson et al. (mean age 67 vs. 71 years). However, this is in line with previous observations suggesting a more rapid progression among older than younger persons with PD [1, 18, 20].

We also found that the treated Later PD cohort with less motor score/symptoms than the de novo PD cohort had worse dynamic balance (TG) and mobility (TUG). TG and TUG focus on more advanced balance in everyday activities such as walking with a narrow base of support, transferring to/from sitting, and turning that are often affected by PD, especially when performed as a sequence [31]. Later PD also presented a higher percentage of individuals with dual-tasking difficulties, and a much higher percentage of those who reported falls/near falls. Also, self-reported walking ability was significantly worse in Later PD in comparison to de novo PD. Altogether, our results support the need of early rehabilitation in addition to dopaminergic medication [16, 46]. Importantly there is also evidence showing that side effects of medication such as dyskinesia has a negative impact on balance and falls in people with PD [9, 35]. In line with this recently established world guidelines for falls prevention and management for older adults highlighted optimization of medications as a critical step to falls prevention in PD treatment [46].

### Clinical implications

The results from testing of various aspects of balance and gait showed that about 25–50% of de novo PD presented values beyond the suggested cutoff scores for increased risk of falling in PD [31, 37]. That implies that early monitoring of balance and gait may be helpful in identifying individuals with early impairments and give the possibility to adapt neurorehabilitation programs based on individual symptom profiles. For example, treadmill training, strategy training (including cueing), balance and gait training, martial arts (Tai Chi and Qigong), and exergaming have been shown to increase gait speed [55]. In patients with mediolateral imbalance, physiotherapists may teach PD patients to widen their step width during gait [22, 30]. In FOG, specific cuing strategies that improve balance and gait may be applied as an alternative treatment to dopaminergic treatment [2, 55]. Exercising also has shown positive effects on reactive balance in PD [49]. The impact of such training on the reduction of falls in PD is also promising [25]. Importantly, key PD trials in rehabilitation and fall prevention showed different results across progression of the disease that may be related to the level of supervision, intensity, or dose of the interventions [8, 47, 48]. For example, less-supervised home-based exercise programs reduce falls in mild PD but not in more sever disease stage [8]. That speaks again for the importance of early and regular monitoring of PD progression preferably provided by a multidisciplinary specialist team.

### Limitations and strengths

Our cohorts were small, and the power of the analyses is, therefore, compromised especially for comparisons between PIGD and non-PIGD subtypes. It might also be of value to have been able to compare the de novo group to a healthy control group to distinguish age differences. In this study, we utilized commonly used clinical tests of balance and gait and found impairments in the de novo group. Interestingly more sensitive “laboratory” methods such as motion analysis with inertial sensors or pressure-sensitive mats have shown similar impairments in balance and gait [12, 29, 32] indicating that these differences can be revealed with clinical assessments but probably more detected with more sensitive methods.

The strength of our study is, however, that both cohorts were consecutively recruited at the same clinic and assessed by the same physiotherapist (BL). However, the recruitment of cohorts did not take place at the same time, but in both cases, the study procedure followed the clinical routines; de novo PD started medication at individual time-points during the 3-month follow-up, and medication adjustment was done when needed in Later PD.

The test battery was selected to cover various aspects of balance and gait and included assessments that are easy and quick to implement in clinical settings. However, the outcome from some of these tests, such as NRT or TG, can vary considerably due to variability in test execution [45, 51]. Therefore, in future studies, it should be of value to use a uniform valid, and reliable assessment that covers various aspects of balance and gait, e.g., Mini-BESTest [41].

## Conclusion

Balance and gait were impaired in de novo PD and most pronounced in PIGD subtype. In addition, dual-task difficulties and falls/near falls were evident during this early stage. The lower scores of motor symptoms in Later PD did not result in better mobility, balance, or less falls/near falls indicating that medications have less effect on these symptoms.

## Data Availability

The data supporting the findings of this study are available on request from the corresponding author. The data are not publicly available due to privacy or ethical restrictions.
